# The Chick as a Model for the Study of the Cellular Mechanisms and Potential Therapies for Alzheimer's Disease

**DOI:** 10.4061/2010/180734

**Published:** 2010-07-18

**Authors:** Radmila Mileusnic, Steven Rose

**Affiliations:** Department of Life Sciences, Faculty of Sciences, The Open University, Milton Keynes, MK7 6AA, UK

## Abstract

While animal experiments have contributed much to our understanding of the mechanisms of Alzheimer's disease (AD), their value in predicting the effectiveness of treatment strategies in clinical trials has remained controversial. The disparity between the results obtained in animal models and clinical trials may in part be explained by limitations of the models and species-specific differences. We propose that one trial passive avoidance in the day-old chick is a useful system to study AD because of the close sequence homologies of chick and human amyloid precursor protein (APP). In the chick, APP is essential for memory consolidation, and disrupting its synthesis or structure results in amnesia. RER, a tripeptide sequence corresponding to part of the growth domain of APP, can restore memory loss and act as a cognitive enhancer. We suggest that RER and its homologues may form the basis for potential pharmacological protection against memory loss in AD.

## 1. Introduction

At first, the day-old chick does not seem a likely model system in which to study the molecular processes involved in a degenerative disease that primarily affects brain function in ageing humans. Before proceeding to argue the case for including the chick amongst such models, however, we should consider more carefully what we mean by, and expect from, an “animal model” of a human disease. 

What does one want from an animal model? By an animal model we mean a nonhuman organism that displays some or all of the features of the human condition we wish to understand. These may include some or all of the genetic, molecular, physiological, anatomical, or behavioural features of the human condition or acceptable analogues thereof. To be of utility, such an organism must be readily amenable to experimental manipulation in one or more of these biological/behavioural levels. As the manifestation of the disease or disorder is likely to be species-typical, especially when we are dealing with neurological or psychiatric diagnoses, inferences as to the relevance of any animal analogue are always going to be problematic, as much of the literature on animal models of depression and schizophrenia has demonstrated [1–5].

Alzheimer's disease (AD) manifests itself in humans in terms of behaviour—initially memory loss and confusion, with progressive decline in other faculties. Neurologically, there is accumulation of amyloid plaques and tangles, neuronal death, notably of cholinergic cells in the hippocampus, and brain shrinkage [6–9]. However, there is no known naturally occurring animal equivalent of these features either behaviourally or neurologically, apart from some partial resemblances such as plaque accumulation in aged captive apes. Thus, animal models have been directed towards mimicking the neurological and/or biochemical features of the disease, primarily in rodents, including transgenics, and examining the behavioural consequences in terms of impaired performance on standard memory tasks [10–12].

## 2. Choice of Task

The measure of neurological deficit commonly chosen as an indicator of an animal model's relevance for AD is an impairment in learning or memory retention in a standard task. Such impairment is taken as analogous to, or better, homologous with, that in human memory in conditions such as AD. Standard laboratory tasks may be aversive or appetitive, single or multiple trial. For rodents, they include passive avoidance and fear conditioning (both single trial) and versions of the Morris water maze (multiple trial). The merit of one trial tasks is that they are sharply timed; the brevity of the training trial allows for a separation of events surrounding the training experience from the processes that occur during memory consolidation. However, single trial learning is not typical of learning in general, because many instances of animal and human learning are based on the acquisition of experience in a number of repeated trials, involving processes such as generalisation, categorisation, and discrimination. Furthermore, the memory expressed in such animal models is procedural rather than declarative, and procedural memory is the last, not the first, to be lost during the degeneration typical of the progression of AD. While it is a necessary assumption for such studies that the biochemical and pharmacological processes explored in the context of animal memory have their parallels in the human case, the repeated failure of agents which act as cognitive enhancers in well-controlled animal experiments to affect human cognition in clinical trials is a salutary warning that the assumption remains-just that.

## 3. The APP and AD

The amyloid precursor protein (APP) is a multifunctional transmembrane glycoprotein involved in diverse and opposing cellular functions such as: synapse formation and maintenance [13–16], memory formation [17–23], regulation of transcription, and neurotoxicity [13, 14]. It is extensively processed posttranslationally by specific proteolytic cleavage [13–15]. Like APP, the APP-derived fragments initiate or execute a variety of cellular functions. Most of the evidence that APP is implicated in memory consolidation is based on the effects of intracerebral or intraventricular injections of exogenous APP, its proteolytic fragments, or antibodies and antisense oligodeoxynucleotides. For example, smaller soluble fragments of *β*-amyloid (A*β*) and structurally mimetic nonpeptidic substances injected centrally antagonise the binding of A*β* protein and produce amnesia [24] as well as a decrease of K^+^-evoked acetylcholine release from hippocampus [25, 26]. Centrally administered amyloid *β* peptides (A*β*) impair retention in the Y-maze, passive avoidance and place-learning in the water maze [26] and cause amnesia for footshock active avoidance in mice [24]. Multiple bilateral injections of A*β*_*1*−40_ into the dorsal hippocampus produce performance decrements in short-term working memory [27]. In contrast to the effects of A*β*, the secreted form of APP (sAPP) is neurotrophic and neuroprotective and when administered intracerebroventricularly, shows potent memory-enhancing effects [26]; amongst other effects it prevents the learning deficits induced by scopolamine in an object recognition task and improves spatial recognition memory [28–30].

APP is generally accepted to be directly involved in AD and consequently has been extensively studied in a number of different mammalian and nonmammalian systems [13, 14]. Thus, attention has been focussed on enzymes such as the alpha, beta, and gamma secretases associated with the misprocessing of APP leading to accumulation of senile plaques and methods for clearing or diminishing plaque load. Animal models for AD such as mice transgenic for the mutant forms of human APP are therefore in principle directed towards any of these processes and events [11]. However, a major weakness of such studies, although very understandable in the earlier days of AD research, has been that the striking appearance of the plaques and the early death of cholinergic cells has focussed excessive attention on these end-products of the biochemical chain of events leading to the disease, on the assumption that they are both proximal causes of the condition and likely therapeutic targets. An alternative hypothesis would be that the primary lesion in the disease is the disruption of neural processes that require the normal functioning of APP and are essential for cognitive functions such as memory. It is towards this hypothesis that our studies in the chick have proceeded, and which in turn has resulted in uncovering a molecular mechanism that could be of therapeutic significance.

## 4. Avian APP

Although birds and mammals diverged about 270 million years ago, and consequently are very different in morphology, behaviour, lifespan, and in the age-dependent repression of a broad-spectrum of neuronal genes, the chick may be a better experimental model to study APP than mice because its APP gene sequence and the enzymatic machinery for processing APP are almost identical to that of humans and closer than those in mice [31–38]. 

Chicken APP expression parallels mammalian APP expression both temporally and topographically. Furthermore, the chick embryo expresses the genes that encode the main proteases implicated in the production of APP, including BACE-1, BACE-2, presenilin-1, presenilin-2, and nicastrin as well as Neprilysin, the main A*β*-degrading enzyme, and ADAM-17, a protease implicated in the nonamyloidogenic processing of APP. Importantly, the level of the APP gene expression is related to the strength of learning in day-old chicks [19]. That makes the chick a useful natural model in which to study the cell biology and functions of APP and a potential “assay system” for drugs that regulate APP processing. 

**Figure 1:**
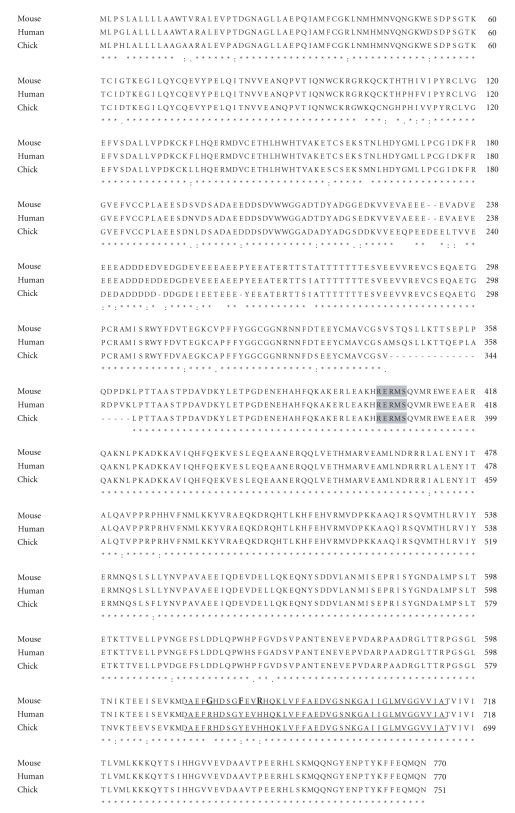
*Alignment of the amino acid sequences of human, mouse, and chick APP.* The numbering refers to the human APP sequence. The RERMS sequence is in gray. Amino acid sequences of A*β* domain are underlined. Residues implicated in amyloidogenesis are indicated in bold. The human (P05067), chicken (Q9DGJ7) and mouse (P12023) APP sequences were obtained from the EMBL database (CLUSTAL 2.0.12 multiple sequence alignment).

The degree of evolutionary conservation of APP is very high. The chick APP gene sequence, similar to that of the mouse, has 93% amino acid identity and 96% similarity with the human sequence (Figure 1). However, it is important to stress that avian A*β* has 100% sequence similarity with the human A*β* sequence in contrast to rodent A*β* which is lacking residues Arg, Tyr, and His in the A*β* domain, shown to be crucial for amyloidogenesis. In addition, the rodent 5′upstream regulatory region of the APP gene is only 82% homologous to the corresponding region of the human APP gene [39]. These differences may be functionally related to the fact that A*β* plaques do not accumulate in aged memory impaired rodents. Another important difference between rodents and humans is related to the sequence of the last 101 C-terminal amino acids of the human APP sequence (corresponding to the A*β*, transmembrane and intracytoplasmic domains). In contrast to mouse and rat, chick and human sequence are identical. That makes the chick a useful natural model in which to study regulation of APP gene expression and the amyloidogenic characteristics of A*β*. 

In contrast to many transgenic models wild type heterozygous chicks do not carry a burden of genetic background which might be a possible confounding factor with regard to crucial aspects of AD [40, 41]. Although sophisticated and precise molecular genetic tools are applied to transgenic animals in order to study the pathophysiology of AD [11, 42, 43], animal performance in the behavioural tests used to assess learning and memory is often affected by variables apparently unrelated to memory function, as shown in an extensive study analysing data from 3003 mice tested in the Morris water maze [44]. This meta-analysis showed that genetic background and environmental differences between laboratories in rearing and handling procedures alone can produce sufficient variation to span the range of most, if not all, behavioural variables and can thus easily mask or fake mutational effects. In addition, disparity attributable to evolutionary divergence between humans and rodents, brings about another type of problem: the strong but incomplete homology between human and mouse APP sequences and the weaker but still considerable homology between APP and APP-like protein (APLP2), compromise accurate measurements of total APP transcript levels in humanised APP transgenic mice and make assessment of the neuropathogenic potential of human APP gene products rather difficult [42, 45, 46].

## 5. The Chick as an Animal Model for the Study of Memory

Our route towards research on AD led through our lab's interest, over many years, in the molecular mechanisms involved in memory storage, on which we have worked extensively with the young chick. The suitability of the chick as a model system for such studies is well documented. Chicks are precocial birds, and need actively to explore and learn about their environment from the moment they hatch. Thus, they learn very rapidly to identify their mother on the basis of visual, olfactory and auditory cues (imprinting), to distinguish edible from inedible or distasteful food, and to navigate complex routes. Training paradigms that exploit these species-specific tasks work with the grain of the animal's biology, and because such learning is a significant event in the young chick's life the experiences involved may be expected to result in readily measurable brain changes. Chicks have large and well-developed brains and soft unossified skulls, making localised cerebral injections of drugs easy without the use of implanted cannulae or anaesthesia. The virtual lack of any blood-brain barrier in these young animals also ensures rapid entry into the brain of peripherally injected agents (for review see [47]).

The training task that we employ is one trial passive avoidance, in which chicks learn to avoid pecking at a small bead coated in the bitter, distasteful, but nontoxic methylanthranilate (MeA). The task has the merits of being rapid and sharply timed (chicks peck a bead within 10 seconds) and as many as 60 chicks can be trained in a single session. Table 1 compares this chick task with those commonly used in rodents. In the standard version of the task in our lab, day-old chicks are held in pairs in small pens, pretrained by being offered a small dry white bead, and those that peck trained with a larger (4 mm dia) chrome or coloured bead coated with MeA. Chicks that peck such a bead show a disgust reaction (backing away, shaking their heads and wiping their bills) and will avoid a similar but dry bead for at least 48 hours subsequently. However, they continue to discriminate, as shown by pecking at control beads of other colours. Chicks trained on the bitter bead are matched with controls which have pecked at a water-coated or dry bead, and which peck the dry bead on test. Generally some 80% of chicks in any hatch group can be successfully trained and tested on this protocol. Each chick is usually trained and tested only once. Because the pecking response requires a positive, accurate act by the bird, it also controls for effects on attentional, visual, and motor processes [47, 48]. 

The training task has two variants: strong, and weak. In the strong version, the aversive substance used to coat the beads is 100% MeA and it produces high and persisting levels of avoidance. However, if the MeA is diluted to 10%, the high level of avoidance for the training bead persists only up 8 hours; long term memories are not formed. In its strong form, the task can be used to identify the molecular cascade involved in memory formation and the interventions that impair consolidation; in its weak form the task can be used to explore potential memory enhancing agents. 

These features make the young chick a highly suitable model for the analysis of the biochemical (and in our hands morphological and physiological) correlates of memory consolidation. Table 1 compares the passive avoidance task in the chick with commonly used tasks in the mouse.

## 6. The Biochemical Cascade of Memory Consolidation in the Chick

Over the past decades a combination of correlative and interventive experimental strategies has enabled us to identify a biochemical cascade that is associated with memory consolidation in the minutes to hours following training. These have been fully reviewed elsewhere [47] so only a brief summary will be given here. In the minutes following training on this task, there is upregulation of N-methyl-D-aspartate receptor activity, phosphorylation of the presynaptic membrane protein B50, and genomic activation of the immediate early genes c-fos and c-jun. During the next hours after training, increased incorporation of fucose into brain glycoproteins occurs. During this time, memory for the passive avoidance task can be impaired by inhibitors of protein and glycoprotein synthesis injected around the time of training. Two regions of the chick brain are involved specifically in the biochemical responses to the learning experience. These are the intermediate medial mesopallium (IMMP), an association “cortical” area, and the medial striatum (MS), a basal ganglia homologue. The chick brain is strongly lateralized and many, but not all, of the molecular events we observe are confined to the left IMMP.

**Figure 2:**
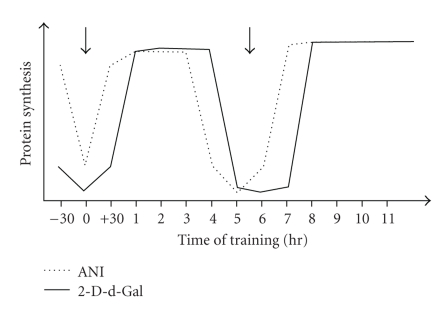
Two time-windows when protein synthesis is sensitive to inhibitors of protein synthesis, such as anisomycin (ANI) and glycoprotein synthesis, such as 2-d-Galactose (2-d-Gal).

All these events depend on de novo protein synthesis and insertion of a variety of proteins, especially glycoproteins, into pre- and postsynaptic membranes. Cell adhesion or cell recognition molecules constitute a major group amongst these glycoproteins. They are expressed both pre- and post-synaptically and involved in the process that allows information about synaptic activity to be simultaneously communicated to both side of the synapse. Our early work identified two such adhesion molecule, L1 and NCAM, which are recruited into this cascade of cellular events at different periods posttraining. Injection of inhibitors of protein and glycoprotein synthesis (anisomycin and 2-deoxy-galactose resp.) at times corresponds to these periods of recruitment (Figure 2) result in amnesia for the task [47]. 

This and related data on the effects of application of protein synthesis inhibitors on memory retention led us to propose that there were two waves of protein synthesis occurring following a learning experience, the first within an hour of the experience and involving the synthesis of proteins expressed by immediate early genes, and the second, some 4–6 hours later, involving structural proteins such as the adhesion molecules. Both are necessary for consolidation of long-term memory.

## 7. APP and Memory Consolidation in the Chick

APP is an important member of the family of cell adhesion molecules, and having identified a role for NCAM and L1 [51–53] in the consolidation cascade, it seemed logical to explore the role of APP itself. Chick APP cross-reacts with the mouse monoclonal antibody raised against human APP. Therefore, we tested the effect of anti-APP antibody on memory. The residence time for the anti-APP corresponds to the relatively rapid turnover time for membrane-bound APP. When injected pre-training, anti-APP did not interfere with the chicks pecking and learning the avoidance task; however, it did result in amnesia in birds tested 30 minutes later. Amnesia persisted for at least the subsequent 24 hours and was not apparent if the antibody was injected just posttraining or 5.5 hours after training [17, 49].

This finding indicated that APP might be required at an early phase and not continually during memory consolidation. Given that blocking APP function by use of specific antibodies outside of a specific time window was without effect, we compromised APP gene expression using antisense oligodeoxynucleotides (AS) designed to correspond to the −146 and to AUG1786 transcription start site of APP [17]. The antisense oligodeoxynucleotides (AS ODNs) were injected intracerebrally at 6 hours or 30 minutes pre-training and chicks were tested at different times posttraining. Injection at 6 hours pre-training was aimed to suppress APP synthesis during the first wave of protein synthesis while the injection made at 30 minutes pre-training was aimed at the second wave (Table 2). Thus, in both groups the AS was present for 6 hours before training. Controls were treated with scrambled (SC) ODNs or saline and trained and tested as the AS ODNs treated groups.

The results showed that APP-antisense both decreased APP gene expression and affected memory formation. The time-window of onset of amnesia relative to time of injection of ODNs and to time of training confirmed our previous finding that APP exerts its function during an early phase of memory formation and appears to be a necessary factor in the biochemical cascade involved in the transition between short- and long-term memory. Our findings on the importance of APP in learning were supported by reports [19] that APP gene expression in the young chick is related to the strength of memory retention for an imprinting task.

## 8. APP-Related Peptides as a Tool to Study Memory

Studies conducted on the physiological activity of APP [54–58] resulted in the identification of a small stretch of amino acids containing the RERMS sequence C-terminal to the KPI insertion site of sAPP-695 as the active domain responsible for growth promotion and neurite extension, neuronal survival, and for sAPP's ability to interfere with the deleterious effects of A*β* on neurons. A synthetic peptide homologous to the RERMS sequence, APP 328–332, was identified as the shortest active peptide to exhibit trophic activity through cell-surface binding and induction of inositol polyphosphate accumulation. Such observations suggested that the RERMS peptide might substitute for sAPP during memory formation and thereby reverse or protect against the blockade resulting from antibody or antisense.

We first assessed the effects of RERMS on memory in chicks rendered amnesic by APP-antisense and APP-antibody treatment [17, 49]. In the series of experiments which followed, we studied the time window in which injection of RERMS might affect amnesia and showed that if injected either before or just after training on the task, the pepide protected against the memory loss. As a control for the behavioural effect of RERMS, we used the reversed peptide sequence SMRER, but to our surprise SMRER was as effective in relieving the memory block as RERMS. However, a different control peptide, RSAER, was without effect. Analysis of these experiments led to two principal observations: first, that the APP-derived peptide might exert its action by compensating for the low presence of APP. According to the proposed amyloid hypothesis, the faulty processing of APP and accumulation of the amyloid fragments might be one of the factors leading to neurotoxicity. Therefore, we tested whether the RERMS peptide might also have a potential protective effect against the memory deficit induced by A*β*. Amyloid-beta, injected into the IMMP bilaterally at a dose of 2 *μ*g/hemisphere, 30 minutes prior to training, resulted in amnesia for the task in chicks tested 24 hours subsequently. However, administration of 1 *μ*g/hemisphere of RERMS 10 minutes after A*β* injection prevented the memory deficit caused by A*β*. Conversely, if the injection is delayed to 5.5 hours posttraining, there is no subsequent amnesia.

**Figure 3:**
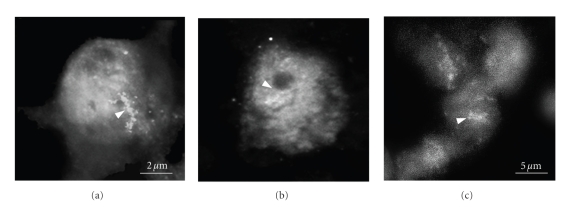
*RER binding detected on chick, human and mouse neuronal cells*. Specific binding of the biotinylated RER (arrows) to chick (a), human (b) and mouse neuronal (c) cell. Location of the chick neuronal cells is in the IMMP area; Human and mouse neuronal cells are located in the CA1 are in hippocampus.

The second observation came from analysis of the amino acid sequence of the peptides used in this study [17, 49]. Both the forward and reverse sequences contain the tripeptide palindrome RER. The next step was therefore to investigate the ability of RER to relieve memory block under the same conditions used for testing RERMS. The RER tripeptide, when injected around the time of training, showed the same potential as the RERMS pentapeptide and rescued memory in animals rendered amnesic by pretreatment with A*β*. We concluded that the RER sequence acts as a core domain of the growth promoting region of APP in the chick because it appears able to substitute for sAPP. The protection against the amnestic effects of A*β* may also result from RER's ability to initiate receptor-mediated processes. This interpretation is strengthened by the evidence that RER binds to two neuronal cell membrane proteins, of ~66 and 110 kDa, respectively. In experiments aimed at identifying specific neuronal binding partners, using a combination of biotinylated tripeptide and cell-specific antibodies, bound RER was localised in chick and human brain sections (Figure 3), suggesting that it might also be active in humans, and could play an important role in the memory formation process which is deficient in the early stages of AD. Moreover, the distribution of biotinylated RER binding suggested membrane binding. In the chick, binding is displaced by longer peptides derived from APP's external domain, but not by A*β*, suggesting that RER competes with sAPP for a putative receptor [17, 49]. 

To overcome the problem of the short half-life of RER we protected it by N-acylation, and showed that Ac-RER is as effective as RER in protecting against memory loss. More importantly, Ac-RER crosses the partially formed blood brain barrier of the one-day old chicks, enabling the peptide to be injected intraperitoneally [17]. The immediate implication of these findings is that it is possible to introduce a behaviourally effective form of RER peripherally by N-terminal acylation, hence protecting the peptide against rapid degradation. 

If the RER sequence acts as a substitute for sAPP than the question to ask is whether it might act as a cognitive enhancer in the weak version of the passive avoidance task discussed above. The weak training protocol is an ideal paradigm to test this hypothesis as memory for the task is not retained beyond an early phase, presumably because the mild aversant does not provide a sufficient signal for the release of sAPP. Under these conditions, tripeptide injected peripherally was as effective as memory enhancer as when injected intracerebrally, meaning that even in the weak training task, in the presence of the tripeptide peptide, memory persists for at least 24 hours. 

All our results point to the short APP-related peptides used in our experiments as both powerful tools in studying the structure and function of APP and as of potential therapeutic interest in AD. We have therefore begun to explore the effectiveness of a number of compounds structurally related to RER. Of particular interest have been the optically isomeric D- or diasteromeric (D/L) forms of the peptide, which are more resistant to proteolysis than their L-equivalents. The diasteromeric forms have begun lately to attract increasing interest as potential immunogens, diagnostic and therapeutic agents [59]. 

**Figure 4:**
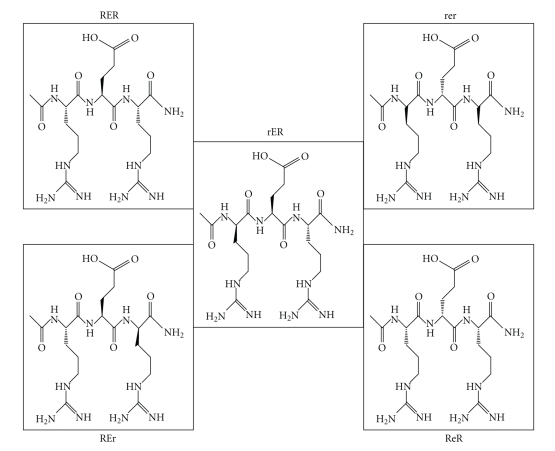
Structure of D/L tripeptides included in the study.

Our results using different D/L forms (shown in Figure 4) demonstrated that substitution of C-terminal L-arginine with the D-isomer essentially abolished the memory retention-enhancing effect of the peptide. This finding pointed to the crucial role of C-terminal L-arginine, in its normal L-conformation, in binding to the peptide's putative receptors. 

Moreover, these experiments clearly show that Ac-rER is a longer lasting and more stable form of the putative memory enhancer than the RER. In addition, it is taken up into the brain from peripheral administration, and is active behaviourally for at least 12 hours following such administration. The fact that there was no difference in the magnitude of the effect of the L- and L/D tripeptide on behaviour suggested that they engage the same biochemical processes [50]. These results are summarised in Table 3.

What is now required is to determine the identity of the RER binding proteins, the specific second messenger systems activated and the genes controlled by RER. Our currently unpublished experiments go some way towards answering these questions, which may be central to understanding the peptide's effects both in memory enhancement and, potentially, in neuroprotection.

## 9. Concluding Remarks

Although it remains important to demonstrate that the peptide or its related structures is effective in other learning tasks and in mammals, we propose that the chick is a useful animal model in which to study AD, and that Ac-rER is a molecule which might form the basis for a potential therapeutic agent in the early stages of AD. Even though some specific details of protein-protein interactions can vary between birds and human, the degree of functional conservation seems to be of particular relevance for the AD field. This animal model, like many other natural model-systems, often appears to suffer from publication bias towards transgenic animal models, which may account for substantial under-representation of avian model system in the experimental literature related to neurodegenerative diseases such as AD.

## Figures and Tables

**Table 1:** Comparison of training tasks in chicks and mice.

	Chicks	Mice
Training paradigm	Passive avoidance	Passive avoidance Fear conditioning Water maze (multiple trials)

Timing	10 secends training time	Passive avoidance - Brief Fear conditioning - Brief Water maze - multiple trials

Suitable for biochemical analysis	Yes	Passive avoidance - yes Fear conditioning - yes Water maze - unsuitable

Sex	Natural distribution of males and females in the hatch. (Sex determined post hoc by inspection of gonads)	Generally males only

Group size	Large (20 chicks/group)	Small

Intracranial injections	Anaesthesia not required	Anaesthesia required

BBB	Not fully developed	Fully developed

Genome	Sequenced in 2004	Sequenced in 2002

Transgenic model	No	Yes, many (for review see Crews et al., 2010; [11])

**Table 2:** Effect of anti-APP and APP antisense on memory retention.

	Time of injection	Memory retention (% Avoidance)	Time of injection	Memory retention (% Avoidance)
Control (Saline, non-immune sera)	30 minutes pre-training	78–95	5.5 hours Posttraining	78–95
Anti APP	30 minutes pre training	28–35**	5.5 hours Posttraining	78–95
Control (SC)	6 hours pre-training	78–95	30 minutes pre-training	78–95
AS	6 hours pre-training	32–37*	30 minutes pre-training	7 8–95

*N* = 18–25; *P < .05; **P < .01.

Anti APP: monoclonal human antibody mAb22C11 [17]; AS: 16-mer end-protected phosphodiester oligodeoxynucleotide, 5′ CXC GAG GAC TGA XCC A 3′, designed to correspond to the transcription start sites −146 and AUG1786 of the *β*APP mRNA, immediately upstream of a ribozyme binding site [17]; SC: Scrambled AS sequence [17]; For further details see [18, 49, 50].

**Table 3:** Summary of peptides and their effects on memory reported in this study.

Peptide	Injection route		Effective dose		Rescue of amnesia induced with:			Enhances weak training	Crossing BBB	*t*1/2 hour
	Ic	ip	Ic *μ*g/brain	Ip mg/kg bw	Anti APP	AS	A*β*			Up to
RERMS	Y	Y	4	20–25	Y	Y	Y	Y	Y	2
SMRER	Y	Y	4	20–25	Y	Y	Y	Y	Y	2
RSAER	N	N	4	/	N	N	N	N	Y	/
RER	Y	Y	4	20–25	Y	Y	Y	Y	Y	/
Ac-RER	Y	Y	16	20–25	Y	/	Y	Y	Y	6
Ac-RRE	N	N	16	/	/	/	N	N	/	/
Ac-rER	Y	Y	16	20–25	Y	/	Y	Y	Y	>12
Ac-REr	N	N	16	/	N	/	N	N	/	/
Ac-ReR	N	N	16	/	N	/	N	N	/	/
Ac-rer	Y	Y	16	20–25	N	/	N	N	/	/

Y: yes, there is an effect on memory; N: No, there is no effect on memory; Anti-APP: monoclonal antibody, clone mAb22C11 [17]; AS: 16-mer end-protected phosphodiester oligodeoxynucleotide, 5′ CXC GAG GAC TGA XCC A 3′, designed to correspond to the transcription start sites −146 and AUG1786 of the *β*APP mRNA, immediately upstream of a ribozyme binding site [17]; A*β*: amyloid-beta. For further details see [18, 49, 50].

## References

[1] Porsolt RD (2000). Animal models of depression: utility for transgenic research. *Reviews in the Neurosciences*.

[2] Kalueff AV, Wheaton M, Murphy DL (2007). What's wrong with my mouse model? Advances and strategies in animal modeling of anxiety and depression. *Behavioural Brain Research*.

[3] Fujii T, Kunugi H (2009). p75NTR as a therapeutic target for neuropsychiatric diseases. *Current Molecular Pharmacology*.

[4] Moisan MP, Ramos A (2010). Rat genomics applied to psychiatric research. *Methods in Molecular Biology*.

[5] Hart PC, Bergner CL, Smolinsky AN (2010). Experimental models of anxiety for drug discovery and brain research. *Methods in Molecular Biology*.

[6] Arnold SE, Hyman BT, Flory J, Damasio AR, Van Hoesen GW (1991). The topographical and neuroanatomical distribution of neurofibrillary tangles and neuritic plaques in the cerebral cortex of patients with Alzheimer’s disease. *Cerebral Cortex*.

[7] Snowden JS, Bathgate D, Varma A, Blackshaw A, Gibbons ZC, Neary D (2001). Distinct behavioural profiles in frontotemporal dementia and semantic dementia. *Journal of Neurology Neurosurgery and Psychiatry*.

[8] Weder ND, Aziz R, Wilkins K, Tampi RR (2007). Frontotemporal dementias: a review. *Annals of General Psychiatry*.

[9] Selkoe DJ (2002). Alzheimer’s disease is a synaptic failure. *Science*.

[10] Götz J, Ittner LM (2008). Animal models of Alzheimer’s disease and frontotemporal dementia. *Nature Reviews Neuroscience*.

[11] Crews L, Rockenstein E, Masliah E (2010). APP transgenic modeling of Alzheimer's disease: mechanisms of neurodegeneration and aberrant neurogenesis. *Brain Structure and Function*.

[12] Bart van der Worp H, Howells DW, Sena ES (2010). Can animal models of disease reliably inform human studies?. *PLoS Medicine*.

[13] Selkoe DJ (1994). Normal and abnormal biology of the *β*-amyloid precursor protein. *Annual Review of Neuroscience*.

[14] Turner PR, O’Connor K, Tate WP, Abraham WC (2003). Roles of amyloid precursor protein and its fragments in regulating neural activity, plasticity and memory. *Progress in Neurobiology*.

[15] Reinhard C, Hébert SS, De Strooper B (2005). The amyloid-*β* precursor protein: integrating structure with biological function. *The EMBO Journal*.

[16] Mucke L (2009). Neuroscience: Alzheimer’s disease. *Nature*.

[17] Mileusnic R, Lancashire CL, Johnston ANB, Rose SPR (2000). APP is required during an early phase of memory formation. *European Journal of Neuroscience*.

[18] Mileusnic R, Lancashire CL, Rose SRR (2004). The peptide sequence Arg-Glu-Arg, present in the amyloid precursor protein, protects against memory loss caused by A*β* and acts as a cognitive enhancer. *European Journal of Neuroscience*.

[19] Solomonia RO, Morgan K, Kotorashvili A, McCabe BJ, Jackson AP, Horn G (2003). Analysis of differential gene expression supports a role for amyloid precursor protein and a protein kinase C substrate (MARCKS) in long-term memory. *European Journal of Neuroscience*.

[20] Doyle E, Bruce MT, Breen KC, Smith DC, Anderton B, Regan CM (1990). Intraventricular infusions of antibodies to amyloid *β*-protein precursor impair the acquisition of a passive avoidance response in the rat. *Neuroscience Letters*.

[21] Huber G, Bailly Y, Martin JR, Mariani J, Brugg B (1997). Synaptic *β*-amyloid precursor proteins increase with learning capacity in rats. *Neuroscience*.

[22] Müller U, Cristina N, Li Z-W (1994). Behavioral and anatomical deficits in mice homozygous for a modified *β*-amyloid precursor protein gene. *Cell*.

[23] Zheng H, Jiang M, Trumbauer ME (1995). *β*-amyloid precursor protein-deficient mice show reactive gliosis and decreased locomotor activity. *Cell*.

[24] Flood JF, Morley JE, Roberts E (1991). Amnestic effects in mice of four synthetic peptides homologous to amyloid *β* protein from patients with Alzheimer disease. *Proceedings of the National Academy of Sciences of the United States of America*.

[25] Abe E, Casamenti F, Giovannelli L, Scali C, Pepeu G (1994). Administration of amyloid *β*-peptides into the medial septum of rats decreases acetylcholine release from hippocampus in vivo. *Brain Research*.

[26] Maurice T, Lockhart BP, Privat A (1996). Amnesia induced in mice by centrally administered *β*-amyloid peptides involves cholinergic dysfunction. *Brain Research*.

[27] Cleary J, Hittner JM, Semotuk M, Mantyh P, O’Hare E (1995). Beta-amyloid(1–40) effects on behavioral and memory. *Brain Research*.

[28] Meziane H, Dodart J-C, Mathis C (1998). Memory-enhancing effects of secreted forms of the *β*-amyloid precursor protein in normal and amnestic mice. *Proceedings of the National Academy of Sciences of the United States of America*.

[29] Bour A, Little S, Dodart J-C, Kelche C, Mathis C (2004). A secreted form of the *β*-amyloid precursor protein (sAPP 695) improves spatial recognition memory in OF1 mice. *Neurobiology of Learning and Memory*.

[30] Ring S, Weyer SW, Kilian SB (2007). The secreted *β*-amyloid precursor protein ectodomain APPs*α* is sufficient to rescue the anatomical, behavioral, and electrophysiological abnormalities of APP-deficient mice. *Journal of Neuroscience*.

[31] Coulson EJ, Paliga K, Beyreuther K, Masters CL (2000). What the evolution of the amyloid protein precursor supergene family tells us about its function. *Neurochemistry International*.

[32] Carrodeguas JA, Rodolosse A, Garza MV (2005). The chick embryo appears as a natural model for research in beta-amyloid precursor protein processing. *Neuroscience*.

[33] Sarasa M, Pesini P (2009). Natural non-trasgenic animal models for research in Alzheimer’s disease. *Current Alzheimer Research*.

[34] Shivers BD, Hilbich C, Multhaup G, Salbaum M, Beyreuther K, Seeburg PH (1988). Alzheimer’s disease amyloidogenic glycoprotein: expression pattern in rat brain suggests a role in cell contact. *The EMBO Journal*.

[35] Yamada T, Sasaki H, Furuya H, Miyata T, Goto I, Sakaki Y (1987). Complementary DNA for the mouse homolog of the human amyloid beta protein precursor. *Biochemical and Biophysical Research Communications*.

[36] Yamada T, Sasaki H, Dohura K, Goto I, Sakaki Y (1989). Structure and expression of the alternatively-spliced forms of mRNA for the mouse homolog of Alzheimer’s disease amyloid beta protein precursor. *Biochemical and Biophysical Research Communications*.

[37] Kang J, Muller-Hill B (1989). The sequence of the two extra exons in rat preA4. *Nucleic Acids Research*.

[38] Kirschner DA, Inouye H, Duffy LK, Sinclair A, Lind M, Selkoe DJ (1987). Synthetic peptide homologous to beta protein from Alzheimer disease forms amyloid-like fibrils in vitro. *Proceedings of the National Academy of Sciences of the United States of America*.

[39] Chernak JM (1993). Structural features of the 5′ upstream regulatory region of the gene encoding rat amyloid precursor protein. *Gene*.

[40] Maynard CJ, Cappai R, Volitakis I (2006). Gender and genetic background effects on brain metal levels in APP transgenic and normal mice: implications for Alzheimer *β*-amyloid pathology. *Journal of Inorganic Biochemistry*.

[41] Wolfer DP, Müller U, Stagliar M, Lipp H-P (1997). Assessing the effects of the 129/Sv genetic background on swimming navigation learning in transgenic mutants: a study using mice with a modified *β*-amyloid precursor protein gene. *Brain Research*.

[42] Heber S, Herms J, Gajic V (2000). Mice with combined gene knock-outs reveal essential and partially redundant functions of amyloid precursor protein family members. *Journal of Neuroscience*.

[43] Kokjohn TA, Roher AE (2009). Amyloid precursor protein transgenic mouse models and Alzheimer’s disease: understanding the paradigms, limitations, and contributions. *Alzheimer’s and Dementia*.

[44] Wolfer DP, Lipp H-P (2000). Dissecting the behaviour of transgenic mice: is it the mutation, the genetic background, or the environment?. *Experimental Physiology*.

[45] Du P, Wood KM, Rosner MH, Cunningham D, Tate B, Geoghegan KF (2007). Dominance of amyloid precursor protein sequence over host cell secretases in determining *β*-amyloid profiles studies of interspecies variation and drug action by internally standardized immunoprecipitation/mass spectrometry. *The Journal of Pharmacology and Experimental Therapeutics*.

[46] Rockenstein EM, McConlogue L, Tan H, Power M, Masliah E, Mucke L (1995). Levels and alternative splicing of amyloid *β* protein precursor (APP) transcripts in brains of APP transgenic mice and humans with Alzheimer’s disease. *The Journal of Biological Chemistry*.

[47] Rose SPR (2000). God’s organism? The chick as a model system for memory studies. *Learning &amp; Memory*.

[48] Gibbs ME, Johnston ANB, Mileusnic R, Crowe SF (2008). A comparison of protocols for passive and discriminative avoidance learning tasks in the domestic chick. *Brain Research Bulletin*.

[49] Mileusnic R, Lancashire CL, Rose SPR (2005). Amyloid precursor protein: from synaptic plasticity to Alzheimer’s disease. *Annals of the New York Academy of Sciences*.

[50] Mileusnic R, Lancashire C, Clark J, Rose SPR (2007). Protection against A*β*-induced memory loss by tripeptide D-Arg-L-Glu-L-Arg. *Behavioural Pharmacology*.

[51] Scholey AB, Mileusnic R, Schachner M, Rose SP (1995). A role for a chicken homolog of the neural cell adhesion molecule L1 in consolidation of memory for a passive avoidance task in the chick. *Learning &amp; Memory*.

[52] Mileusnic R, Rose SPR, Lancashire C, Bullock S (1995). Characterisation of antibodies specific for chick brain neural cell adhesion molecules which cause amnesia for a passive avoidance task. *Journal of Neurochemistry*.

[53] Mileusnic R, Lancashire C, Rose SPR (1999). Sequence-specific impairment of memory formation by NCAM antisense oligonucleotides. *Learning &amp; Memory*.

[54] Ohsawa I, Takamura C, Kohsaka S (1997). The amino-terminal region of amyloid precursor protein is responsible for neurite outgrowth in rat neocortical explant culture. *Biochemical and Biophysical Research Communications*.

[55] Li HL, Roch J-M, Sundsmo M (1997). Defective neurite extension is caused by a mutation in amyloid *β*/A4 (A*β*) protein precursor found in familial Alzheimer’s disease. *Journal of Neurobiology*.

[56] Jin L-W, Ninomiya H, Roch J-M (1994). Peptides containing the RERMS sequence of amyloid *β*/A4 protein precursor bind cell surface and promote neurite extension. *Journal of Neuroscience*.

[57] Yamamoto K, Miyoshi T, Yae T (1994). The survival of rat cerebral cortical neurons in the presence of trophic APP peptides. *Journal of Neurobiology*.

[58] Ninomiya H, Roch J-M, Sundsmo MP, Otero DAC, Saitoh T (1993). Amino acid sequence RERMS represents the active domain of amyloid *β*/A4 protein precursor that promotes fibroblast growth. *Journal of Cell Biology*.

[59] Fujii N (2002). D-amino acids in living higher organisms. *Origins of Life and Evolution of the Biosphere*.

